# Evidence of the niche expansion of crofton weed following invasion in China

**DOI:** 10.1002/ece3.9708

**Published:** 2023-01-06

**Authors:** Xiaoqing Xian, Haoxiang Zhao, Rui Wang, Hongbin Zhang, Baoxiong Chen, Wanxue Liu, Fanghao Wan

**Affiliations:** ^1^ State Key Laboratory for Biology of Plant Diseases and Insect Pests Institute of Plant Protection, Chinese Academy of Agricultural Science Beijing China; ^2^ Rural Energy and Environment Agency Ministry of Agriculture and Rural Affairs Beijing China

**Keywords:** *Ageratina adenophora*, invasive alien plants, MaxEnt, niche dynamics, potential geographical distribution

## Abstract

Niche dynamics of invasive alien plants (IAPs) play pivotal roles in biological invasion. *Ageratina adenophora*—one of the most aggressive IAPs in China and some parts of the world—poses severe ecological and socioeconomic threats. However, the spatiotemporal niche dynamics of *A. adenophora* in China remain unknown, which we aimed to elucidate in the present study. China, Mexico; using a unifying framework, we reconstructed the climate niche dynamics of *A. adenophora* and applied the optimal MaxEnt model to predict its potential geographical distribution in China. Furthermore, we compared the heterogeneity of *A. adenophora* niche between Mexico (native) and China (invasive). We observed a low niche overlap between Mexico (native) and China (invasive). Specifically, the niche of *A. adenophora* in China has distinctly expanded compared to that in Mexico, enhancing the invasion risk of this IAP in the former country. In fact, the climatic niche of *A. adenophora* in Mexico is a subset of that in China. The potential geographical distribution of *A. adenophora* is concentrated in the tropical and subtropical zones of Southwest China, and its geographical distribution pattern in China is shaped by the combination of precipitation and temperature variables. The niche dynamics of *A. adenophora* follow the hypothesis of niche shift and conservatism. The present work provides a unifying framework for studies on the niche dynamics of other IAPs worldwide.

## Graphical abstract

Using a unifying framework, we reconstructed the climate niche dynamics of *A. adenophora* and applied the optimal MaxEnt model to predict its potential geographical distribution in China. Furthermore, we compared the heterogeneity of its niche between Mexico (native) and China (invasive).

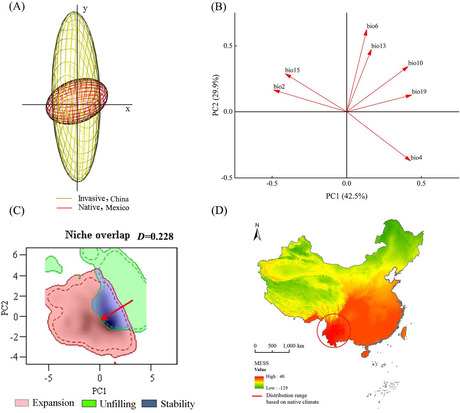

## 1 INTRODUCTION

Invasive alien plants (IAPs) represent a severe global issue in the Anthropocene, posing widespread ecological (i.e., related to biodiversity and ecosystem) and socioeconomic (i.e., related to the natural environment and human health) risks (Bartz & Kowarik, 2019; Carboni et al., 2021; Kumar Rai & Singh, 2020; Livingstone et al., 2020). In this light, invasion risk assessments of IAPs have gradually become a global research hotspot in invasion biology (Adhikari et al., 2021; Chen et al., 2015; Fridley et al., 2021). The spatiotemporal niche dynamics of IAPs, including niche conservatism and shifts, are the key assumptions for assessing their invasion risk (Broennimann et al., 2007; Pearman et al., 2008; Wiens et al., 2010) as well as elucidating their distribution patterns (Wiens & Graham, 2005), adaptations to diverse environmental variables (Ørsted & Ørsted, 2019; Petitpierre et al., 2012), and mechanisms underlying invasion processes worldwide (Atwater et al., 2018; Tingley et al., 2014). Recent studies have explored the niche dynamics, specifically niche conservatism and shifts, of IAPs during global invasion processes (Atwater et al., 2018; Liu et al., 2020a). Niche conservatism over the course of evolution was first proposed in 1999 (Peterson et al., 1999). Subsequently, in the 2000s, some evidence of niche shifts of invasive alien species (IAS) during biological invasion was gathered (Broennimann et al., 2007). Since the 2010s, quantitative evolutionary studies of the niche dynamics of IAS emerged (Chapman et al., 2017; Datta et al., 2019; MacDougall et al., 2009). Based on previous research, niche shift has been proposed to be a common phenomenon during the invasion process of IAPs worldwide (Atwater et al., 2018). Previous studies showed that the adaptive evolution of IAPs' traits and their rapid genetic adaptation increased their climatic tolerances, which in turn changed the fundamental niche (Sotka et al., 2018). However, more experiments are needed to demonstrate whether rapid adaptation of IAPs is a frequent occurrence. Therefore, niche conservatism and shifts of IAPs across space and time remain debatable. In this context, elucidating the presence of niche dynamics of IAPs is pivotal for formulating effective conservation strategies.

Approaches and findings regarding the niche dynamics of IAS remain largely controversial. In 2014, a unifying framework with two approaches commonly employed to quantify the niche dynamics of IAS between invasive and native ranges, including climate niches with observational data and spatial predictions using species distribution models (SDMs), was proposed (Guisan et al., 2014). Specifically, the Centroid shift, Overlap, Unfilling, and Expansion (COUE) scheme and environmental principal component analysis (PCA‐env) (Broennimann et al., 2012) have been used to analyze climate niche shifts of common ragweed (*Ambrosia artemisiifolia* L.) in Europe (Chapman et al., 2017), broad‐leaf privet (*Ligustrum lucidum* W. T. Aiton) worldwide (Dreyer et al., 2019), and 815 terrestrial IAPs introduced across five continents (Atwater et al., 2018). Furthermore, SDMs, which are reliable and powerful tools, have been applied for spatial predictions of IAS based on occurrence data and environmental variables (Lake et al., 2020; Liu et al., 2020b). Among these, MaxEnt model is one of the most frequently used models for predicting the potential geographical distributions of IAS and has been increasingly used in recent years (Lantschner et al., 2019). MaxEnt model can solve the complicated interactions between predicted factors and species distribution data, and also has good performance in previous studies (Elith et al., 2011). For instance, MaxEnt was used to study the dynamics of the geographical distribution of IAPs in western Himalayas (Thapa et al., 2018), quantifying the invasion risk of 896 terrestrial IAPs in the United States under climate change (Allen & Bradley, 2016), and determining the potential geographical distribution of 13 IAPs worldwide (Wan et al., 2019). Currently, the unifying framework is frequently used to study the niche dynamics of IAPs and assess whether these dynamics accelerate invasion processes worldwide.

*Ageratina adenophora* (Spreng.) R.King & H.Rob., known as Crofton weed, is a very aggressive IAP native to Mexico (Auld & Martin, 1975). It is widely distributed in Asia, Oceania, Africa, and Europe and has gradually become a significant IAP worldwide (Poudel et al., 2019). *Ageratina adenophora* is a destructive weed in Australia (New South Wales and Queensland) and the United States (Florida and Hawaii) (CABI, 2022). In China, *A. adenophora* was listed as a key management IAS under the administration of the Ministry of Agriculture and Rural Affairs, the Ministry of Natural Resources, and the Ministry of Ecology and Environment. Following the successful invasion of Yunnan (southwestern China) in the 1940s, *A. adenophora* rapidly spread to southwest China, including Yunnan, Sichuan, Chongqing, Guizhou, and Guangxi (Sang et al., 2009; Wang & Wang, 2006). In China, *A. adenophora* has caused serious ecological problems and economic losses, such as reducing the local biodiversity (Chandra et al., 2019; Lu et al., 2006); destroying the ecological community structure (Fu et al., 2018); altering the soil microbial community structure (Li et al., 2022); and impeding the development of agriculture, forestry, and animal husbandry (Poudel et al., 2019; Xu et al., 2006; Yan et al., 2001). The area of farmland, forest, and grassland occupied by *A. adenophora* accounted for 8.77%, 29.31%, and 18.52% of the total area in Guizhou, resulting the direct economic loss reached 480 million China yuan (Long et al., 2011). *Ageratina adenophora* has the strong reproductive ability. For sexual reproduction, its seeds are huge. Each plant can produce 30–45 thousand seeds, and some even more than 100 thousand seeds, which can be dispersed by wind, water, and the movement of humans and animals (Li et al., 2017; Wang et al., 2011). For asexual reproduction, the rhizomes of *A. adenophora* can be asexually reproduced (Li et al., 2017). All the above characteristics make it more difficult to be managed. Previous studies on *A. adenophora* mainly focused on prevention and control measures (Kluge, 1991; Poudel et al., 2019), niche expansion at the intercontinental scale (Datta et al., 2019), allelopathy (Jiao et al., 2021), and biological characteristics (Zheng et al., 2018); however, the niche dynamics of *A. adenophora* in China remain unknown. The niche dynamics of *A. adenophora*, including its climate niche conservatism and shift as well as changes in its geographical distribution, are closely linked to the geographical distribution pattern and spread of this IAP in China. Therefore, clarifying the niche dynamics of *A. adenophora* can guide its prevention and control. To this end, based on the unifying framework, the present study aimed to address the following issues. We first reconstructed the climate niche dynamics of *A. adenophora* in China and compared the heterogeneity of its climate niches between Mexico and China. Next, based on the optimal MaxEnt model combined with occurrence data and bioclimatic variables, we predicted the potential geographical distribution of *A. adenophora* in China and identified significant bioclimatic variables shaping its geographical distribution. The combined unifying framework allowed us to gain a better understanding of the dynamics of climate niche and geographical distribution pattern of *A. adenophora* during the invasion process and identify its further invasion in China. Our findings provide a unified reference framework for predicting the niche dynamics of other IAPs in the world.

## 2 METHODS

### 2.1 Occurrence data of *Ageratina adenophora*

**FIGURE 1:**
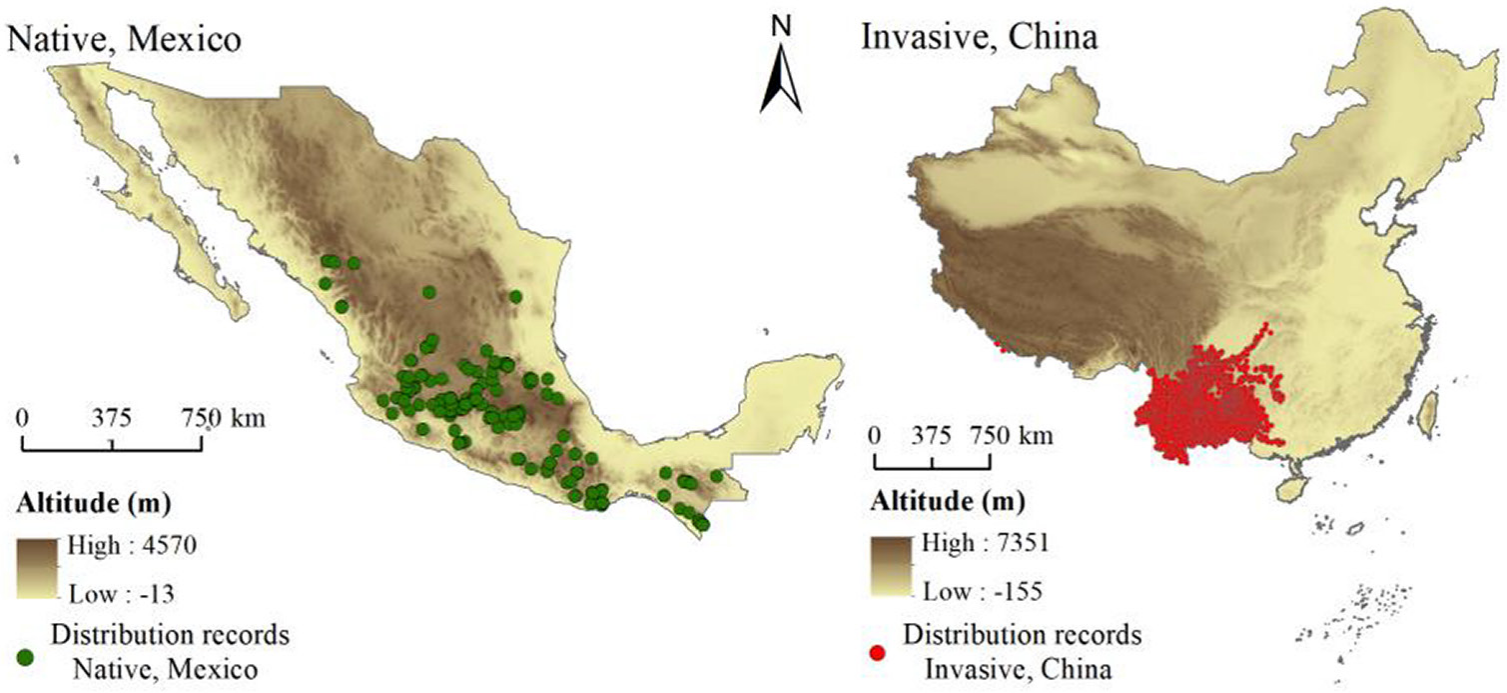
Occurrence data of *Ageratina adenophora* in its native (Mexico) and invasive (China), countries. The data were retrieved from online databases and field surveys.

Occurrence data of *A. adenophora* in the native (Mexico) and invasive (China) countries were collected and interpreted. First, we searched the occurrence data of *A. adenophora* from online databases, including the Southwest Environmental Information Network (SEINet, http://swbiodiversity.org), Global Biodiversity Information Facility (GBIF, http://www.gbif.org/), and Chinese Virtual Herbarium (CVH, http://www.cvh.ac.cn) and our field survey data. Further, we obtained detailed occurrence data of *A. adenophora* in China through field surveys. Next, we saved occurrence data with detailed geographic information and used ENMTools to assign the occurrence data of *A. adenophora* such that only one occurrence point was retained per grid (5 × 5 km) to minimize the bias or error (regarding the resolution of environmental variables) (Warren et al., 2010). Finally, 224 occurrence points in Mexico and 2481 occurrence points in China were obtained (Figure 1).

### 2.2 Environmental variables

From the WorldClim database (http://www.worldclim.org/), environmental variables, including 19 global bioclimatic variables during 1970–2000, were obtained (Table S1). The collinearity between environmental variables often leads to overfitting of the MaxEnt model. Using ENMTools, we performed a correlation analysis of 19 bioclimatic variables to eliminate multivariate collinearity (Figures S1 and S2). Meaningful bioclimatic variables (|*r*| < 0.8) were retained. Seven bioclimatic variables were retained for Mexico: mean diurnal range (Bio2), temperature seasonality (standard deviation ×100) (Bio4), minimum temperature of the coldest month (Bio6), mean temperature of the warmest quarter (Bio10), precipitation of the wettest month (Bio13), precipitation seasonality (coefficient of variation) (Bio15), and precipitation of the coldest quarter (Bio19). Six bioclimatic variables were retained for China: isothermality (Bio3), maximum temperature of the warmest month (Bio5), minimum temperature of the coldest month (Bio6), annual temperature range (Bio7), annual precipitation (Bio12), and precipitation seasonality (coefficient of variation) (Bio15).

### 2.3 Climate niche of *A. adenophora*

Seven bioclimatic variables (Bio2, Bio4, Bio6, Bio10, Bio13, Bio15, and Bio19) in Mexico, as screened by ENMTools, were used to analyze the climatic niche of *A. adenophora*. The climate niche space of *A. adenophora* was constructed in three steps. First, the whole climate niche spaces of *A. adenophora* in Mexico and China were compared and mapped in n‐dimensional environmental spaces using Niche Analyst (Qiao et al., 2016). Then, climate niche dynamics based on occurrence and bioclimatic data were compared between Mexico and China using the COUE scheme and analytical framework (Broennimann et al., 2012). Seven available bioclimatic variables (Bio2, Bio4, Bio6, Bio10, Bio13, Bio15, and Bio19) were used to perform PCA‐env and generate predicted niche occupancy profiles. Next, climate niche equivalency and similarity tests were run in both directions (Mexico↔China) using 100 repetitions. If the value of the observed climate niche was significantly lower than the value of the climate niche overlap (*p* < .05), the null hypothesis of climate niche equivalency was rejected, while the observed niche overlap was more similar to each other than expected by chance (Warren et al., 2008). Schoener's *D* was used to assess the similarity of *A. adenophora* climate niches between Mexico and China (Schoener, 1968). The *D* value employs metrics ranging from 0 (indicating no overlap) to 1 (>0.6 indicating significant overlap). All analyses were performed using the “ecospat” package in R 4.1.2 (Di Cola et al., 2017). Finally, multivariate environmental similarity surface (MESS) was used to compare the climate niches of *A. adenophora* between Mexico and China. The smaller the *S* value, the greater the climatic difference. An *S* value of 100 indicates a lack of differences in environmental variables (Elith et al., 2010). We used “density.tools.Novel” to analyze MESS in MaxEnt.

### 2.4 Potential geographical distribution of *A. adenophora*

MaxEnt was used to simulate the potential geographical distribution of *A. adenophora* through three steps. First, the R package “ENMeval” was used to calibrate the MaxEnt model by setting different combinations of feature classes (FCs: linear‐L, quadratic‐Q, product‐P, threshold‐T, and hinge‐H) and regularization multiplier (RM) (Kass et al., 2021). RM was set gradually from 0.5 to 4 at 0.5 intervals, and six combinations of FCs, including “L,” “LQ,” “H,” “LQH,” “LQHP,” and “LQHPT,” were used. Finally, 48 models were tested, including the default auto‐feature model. The optimal MaxEnt model with the smallest delta AICc (Akaike information criterion) value was selected. Next, for the optimal MaxEnt model, 25% of the occurrence records were used for testing and the remaining 75% were used for training. The maximum number of background points was set to 10,000. The accuracy of the MaxEnt model was evaluated using the area under the receiver operating characteristic (ROC) curve (AUC). The ROC curve is a type of acceptance curve in which the horizontal and vertical coordinates represent false‐ and true‐positive rates, respectively (Peterson et al., 2008). The higher the AUC value, the higher the accuracy of the MaxEnt model outputs. Finally, the maximum value of the MaxEnt model outputs with 10 replicates was selected to obtain the final results of the present study. Suitable habitats of *A. adenophora* were converted to raster format, ranked, and extracted using the Chinese administrative division map in ArcGIS. The suitable habitats were classified into four types based on the minimum training presence (MTP): high‐suitability habitat (0.5 ≤ *p* ≤ 1.0), moderate‐suitability habitat (0.3 ≤ *p* < .5), low‐suitability habitat (MTP ≤ *p* < .3), and unsuitable habitat (0.0 ≤ *p* < MTP).

## 3 RESULTS

### 3.1 FCs and RM of the optimal MaxEnt model

**FIGURE 2:**
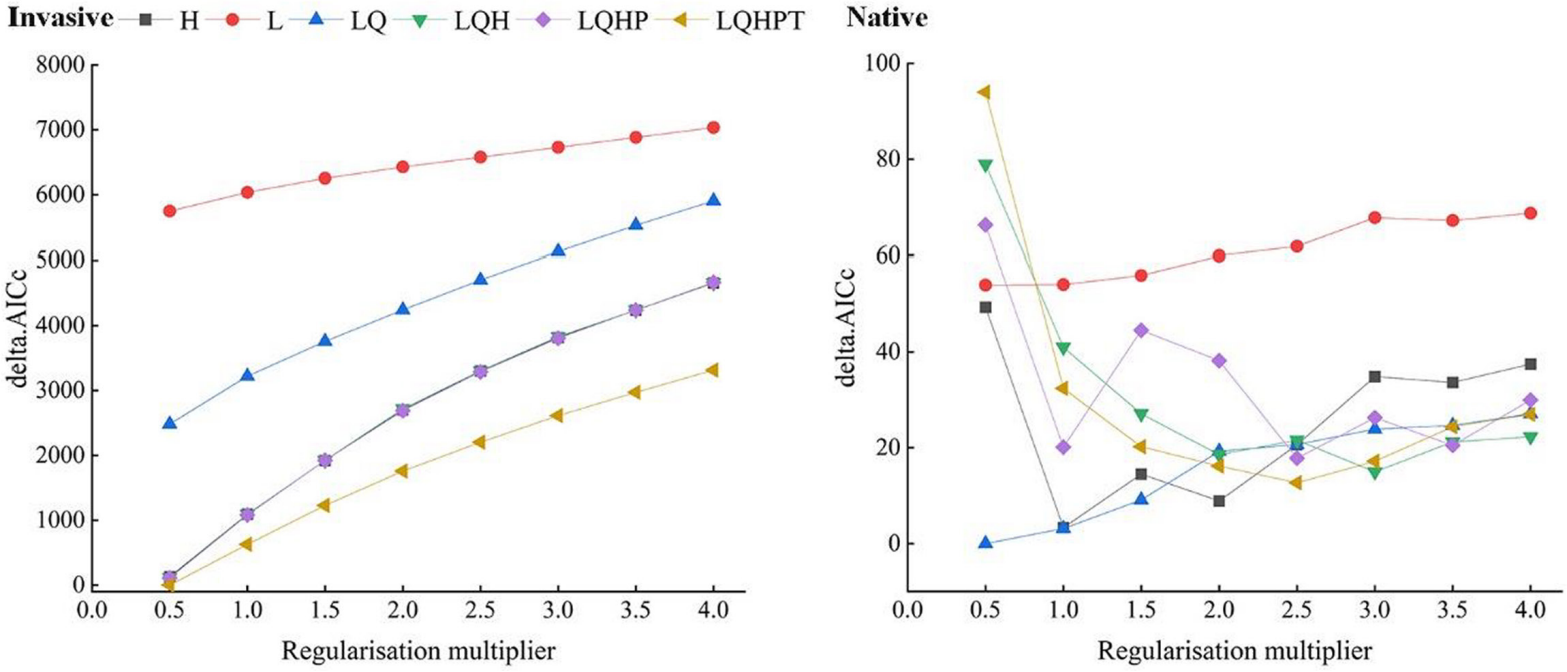
Delta AICc values of the candidate models for *Ageratina adenophora*. Invasive: Based on bioclimatic variables and occurrence records in China. Native: Based on bioclimatic variables and occurrence records in Mexico.

The results of MaxEnt model calibration are presented in Figure 2. Optimal MaxEnt models were selected based on the smallest delta AICc values. Based on the bioclimatic variables and occurrence records of *A. adenophora* in China (invasive), the FCs were LQHPT and RM was 0.5 in the optimal MaxEnt model. Based on the bioclimatic variables and occurrence records of *A. adenophora* in Mexico (native), the FCs were LQ and the RM was 0.5 in the optimal MaxEnt model. Moreover, the mean AUC values in the optimal MaxEnt model based on the bioclimatic variables and occurrence records of *A. adenophora* in China (invasive) and Mexico (native) were 0.893 and 0.899, respectively (Figure S3), indicating good prediction accuracy of the model output.

### 3.2 Climate niche overlap, MESS, equivalency, and similarity

**FIGURE 3:**
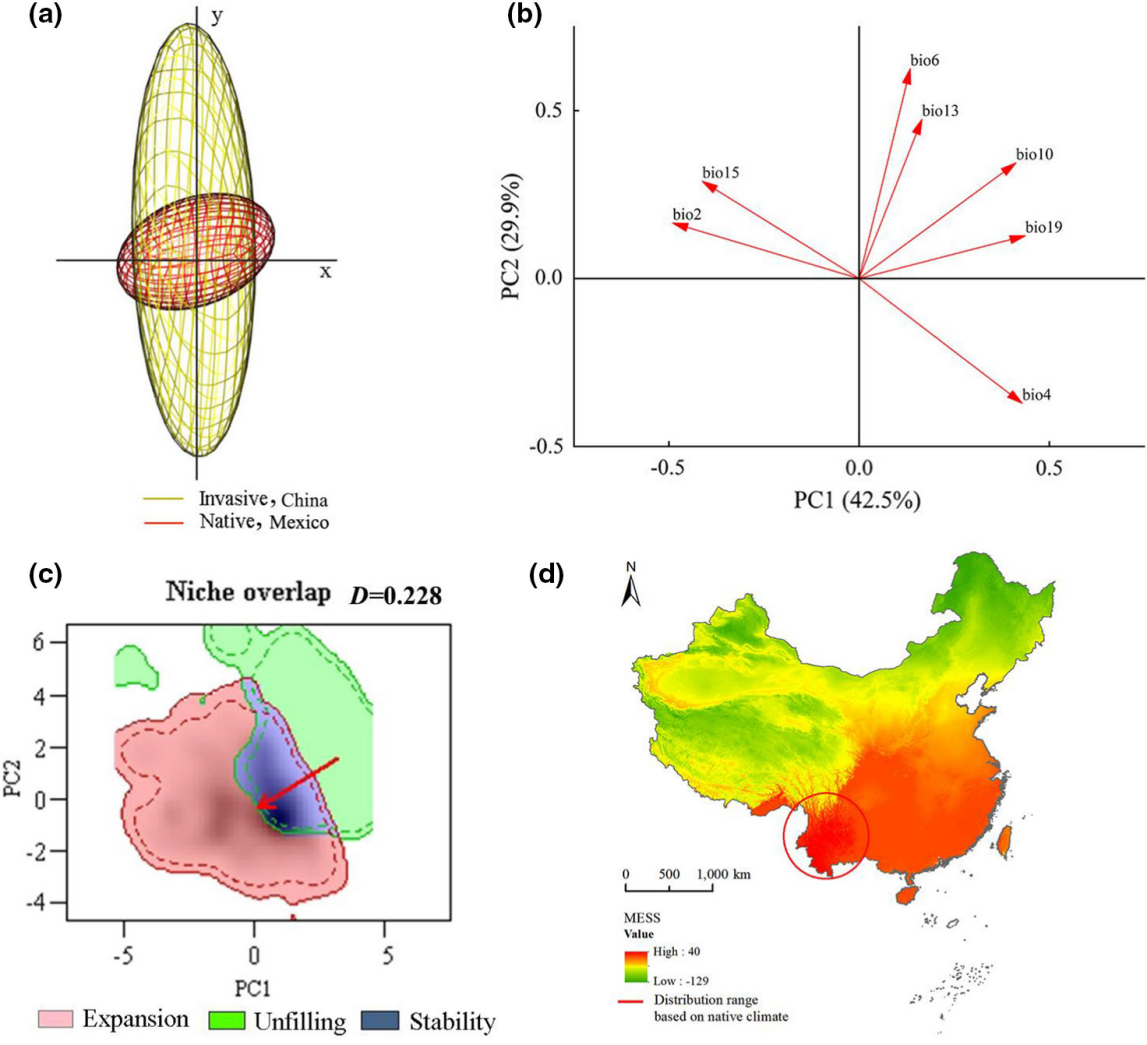
Niches of *Ageratina adenophora* in Mexico (native) and China (invasive) under the climatic space. (a) The whole climate space of *A. adenophora*. (b) Contribution rates of bioclimatic variables on two principal component axes (PC1 and PC2). (c) Climate niche overlap of *A. adenophora*. (d) Analysis of multivariate environmental similarity surface.

The climate niche dynamics of *A. adenophora* in China based on the comparison of climate niche space between Mexico and China are presented in Figure 3. A greater extent of climate niche space of *A. adenophora* in China was unoccupied than that in Mexico (Figure 3a). The first two principal components (PC1 and PC2) explained 72.4% of the total variation in bioclimate variables (PC1 = 42.5%, PC2 = 29.9%). Specifically, precipitation and temperature variables jointly affected the geographical distribution pattern of *A. adenophora* (Figure 3b). Climate niche overlap based on occurrence data and bioclimatic variables in Mexico and China revealed a Schoener's *D* of 0.228, indicating a low degree of overlap. The climate niche in China is expanded compared with that in Mexico (Figure 3c). MESS analysis revealed a highly similar zone of *A. adenophora* climate niche between Mexico and China at the edge of the tropical zone, south subtropical zone, and middle subtropical zone in China (Figure 3d). The potential geographical distribution of *A. adenophora* in China estimated based on bioclimatic variables and occurrence data in Mexico using the optimal MaxEnt model also revealed a highly similar zone of the climate niche in China at the same location.

**FIGURE 4:**
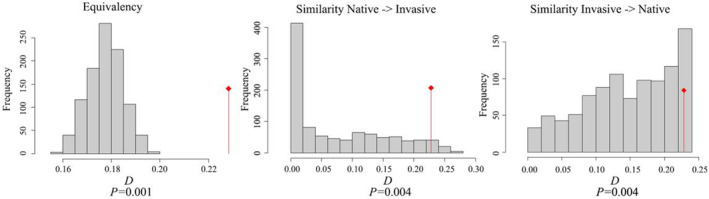
Niche equivalency and similarity between Mexico (native) and China (invasive). Red arrows indicate Schoener's *D*.

The climate niche of *A. adenophora* based on the bioclimatic variables of Mexico and China was not equivalent (*p* = .001) (Figure 4). Regarding climate niche similarity of *A. adenophora* in both directions (Mexico↔China), all *p* values were .004, indicating that the observed niche was more similar to each other than expected by chance (Figure 4). Therefore, *A. adenophora* has undergone alterations in its climate niche during its invasion process.

### 3.3 Predicted niche occupancy (PNO) profiles between Mexico (native) and China (invasive)

**FIGURE 5:**
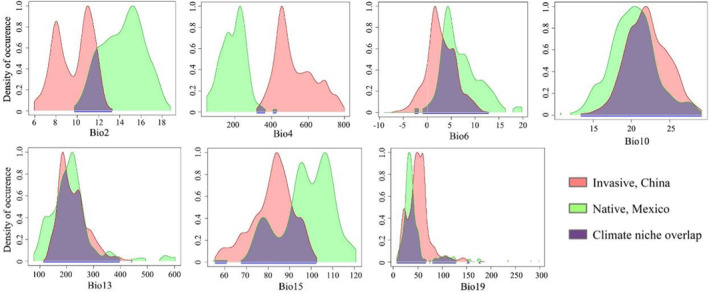
Predicted niche occupancy profiles. Bio2: Mean diurnal range. Bio4: Temperature seasonality (standard deviation ×100). Bio6: Minimum temperature of the coldest month. Bio10: Mean temperature of the warmest quarter. Bio13: Precipitation of the wettest month. Bio15: Precipitation seasonality (coefficient of variation). Bio19: Precipitation of the coldest quarter.

Predicted niche occupancy profiles revealed heterogeneity in the climatic requirements of *A. adenophora* populations in native (Mexico) and invasive (China) countries (Figure 5). Regarding temperature requirements, including the mean diurnal range (Bio2), temperature seasonality (standard deviation ×100) (Bio4), minimum temperature of the coldest month (Bio6), and mean temperature of the warmest quarter (Bio10), invasive (China) *A. adenophora* populations showed better suitability to lower temperatures than native (Mexico) populations. Regarding precipitation requirements, including precipitation of the wettest month (Bio13), precipitation seasonality (Bio15), and precipitation of the coldest quarter (Bio19), invasive (China) *A. adenophora* populations showed better suitability to higher precipitation in the wettest month and coldest quarter but a lower coefficient of variation for precipitation seasonality than native (Mexico) populations.

### 3.4 Potential geographical distribution of *A. adenophora* in native and invasive countries

**FIGURE 6:**
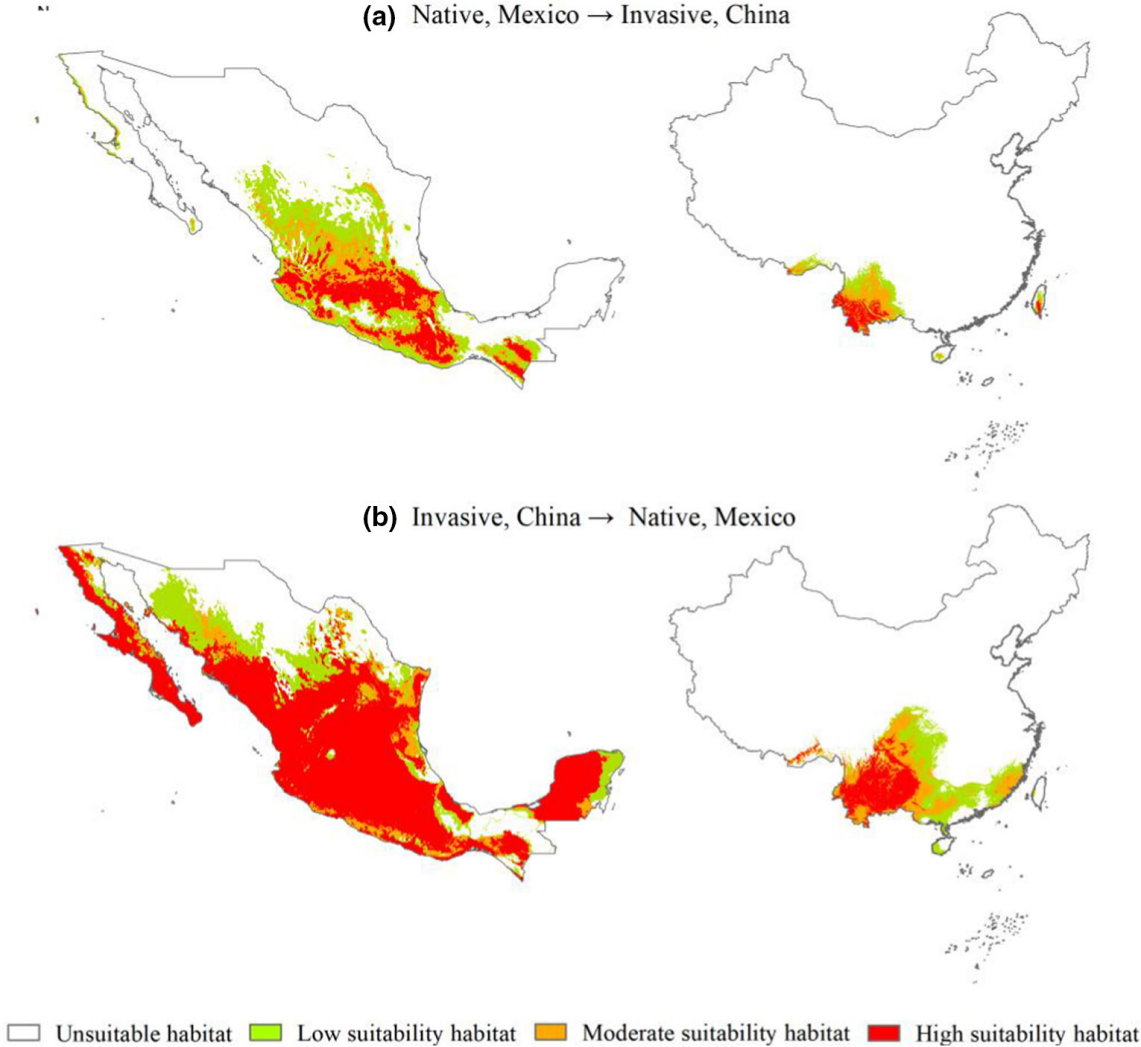
Potential geographical distribution of *Ageratina adenophora* in Mexico and China based on the corresponding bioclimatic variables and occurrence records. (a) Based on bioclimatic variables and occurrence records in Mexico. (b) Based on bioclimatic variables and occurrence records in China.

Reciprocal prediction of the potential geographical distribution of *A. adenophora* in Mexico (native) and China (invasive) further validated its niche expansion (Figure 6). However, the optimal MaxEnt model for *A. adenophora* fitted in Mexico failed to predict the potential geographical distribution of *A. adenophora* in China, and vice versa, regardless of the modeling technique or meteorological data collection. The results of the optimal MaxEnt model for *A. adenophora* fitted in Mexico predicted the potential geographical distribution of *A. adenophora* in China at the edge of the tropical zone and in the western part of the middle and northern subtropical zones. The high‐suitability habitats were concentrated at the edge of the tropical and northern subtropical zones of China. Meanwhile, moderate‐ and low‐suitability habitats were concentrated in the middle subtropical zone of China. The areas of the potential geographical distribution of *A. adenophora* in Mexico were concentrated in the central, southeastern, and southern zones. The results of optimal MaxEnt model for *A. adenophora* fitted in China predicted that the areas of the potential geographical distribution of this IAP are spread at high latitudes in northern subtropical zones of China and high latitude zones in Mexico.

### 3.5 Potential geographical distribution and spread of *A. adenophora* in China

**FIGURE 7:**
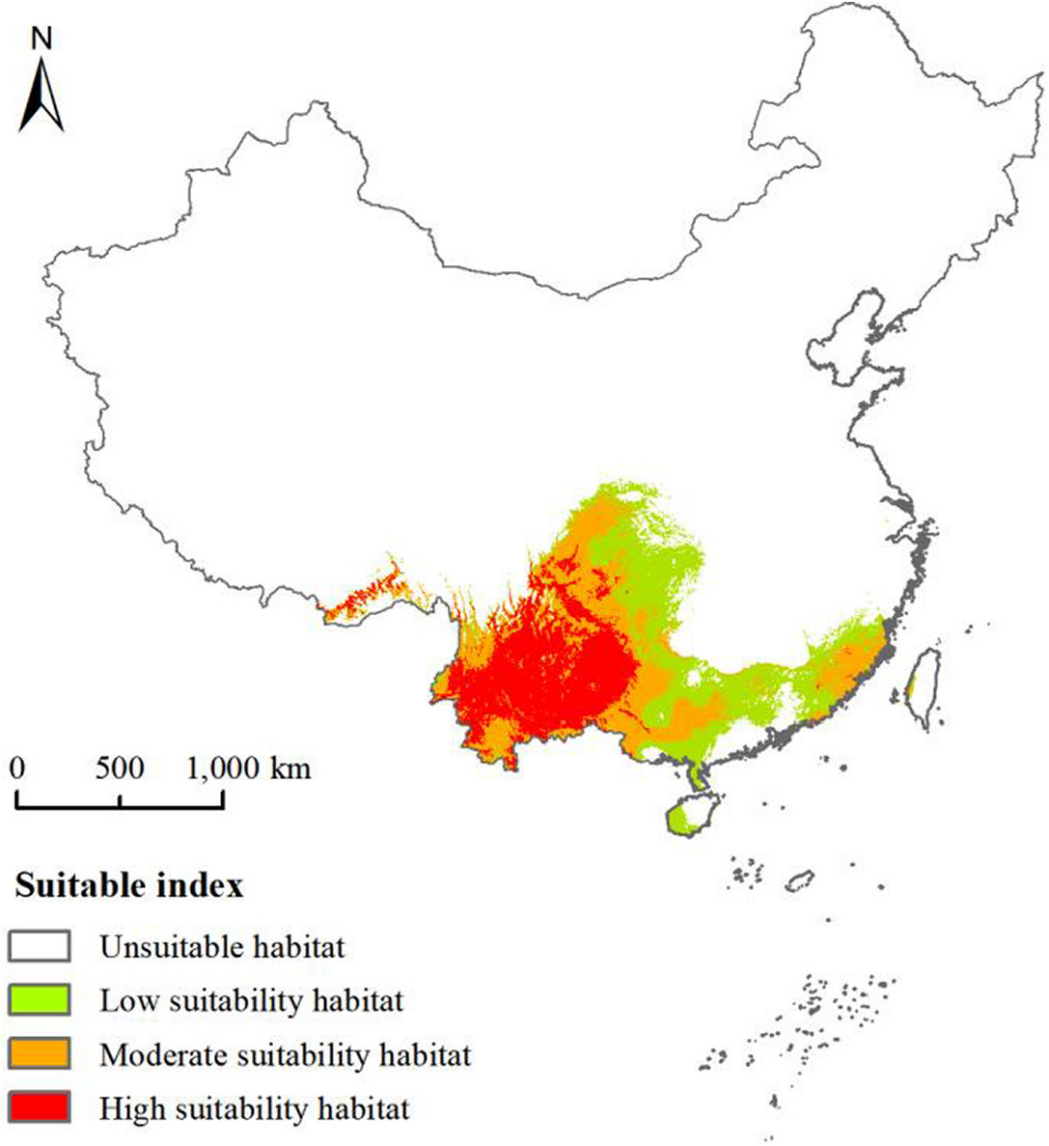
Potential geographical distribution of *Ageratina adenophora* in China.

The potential geographical distribution and spread trends of *A. adenophora* are presented in Figure 7. The total‐suitability habitats covered an area of 126.10 × 10^4^ km^2^ and were primarily distributed in southern and south‐eastern China across the five temperature zones. Further, the high‐suitability habitats covered an area of 38.17 × 10^4^ km^2^ and were mainly distributed in the middle and south subtropical zones and at the edge of the tropical zone. Moreover, the moderate‐suitability habitats covered an area of 42.91 × 10^4^ ha and were mainly distributed in the north and middle subtropical zones and at the edge of the tropical zone. The potential geographical distribution range of *A. adenophora* spanned from tropical to subtropical zones during the invasion process in China. The areas of potential geographical distribution harbor many rivers and water systems. *Ageratina adenophora* seeds can naturally drift over long distances along rivers. Therefore, river and water systems have a great impact on the dispersal of *A. adenophora*.

## 4 DISCUSSION

### 4.1 Significance of niche dynamic analysis

The niche of IAPs, linking their geographical distribution with abiotic and biotic factors in the natural environment (MacDougall et al., 2009; Wiens et al., 2009), represents a major research topic in ecology and evolution studies (Gong et al., 2020; Higgins & Richardson, 2014). Climate is an important part of the species' niche. Factors other than bioclimatic variables can also influence the distribution of the IAPs. For instance, the biotic variables (competition, presence or absence of predation risk, and dispersal ability) can limit the geographical distribution of species (Abdulwahab et al., 2019; Maynard et al., 2017). Assessing the niche dynamics of IAPs across different invasion countries or periods in the face of climate change can elucidate the invasion risk of IAPs and their spread during the process of invasion. For instance, niche shifts of IAPs may increase their invasive potential at the regional scale (Christina et al., 2019). Multiple factors have significantly contributed to the rapid expansion of *A. adenophora* in southwestern China (Wang et al., 2011). However, whether *A. adenophora* niche has shifted during its invasion process in China remains unclear.

In the present study, we focused on selecting the bioclimatic variables to facilitate the comparison of the niche of *A. adenophora* in native (Mexico) and invasive (China) owing to the importance of that on species distributions. We used a previously proposed framework (Guisan et al., 2014) to reconstruct the climate niche space and potential geographical distribution of *A. adenophora* in its native (Mexico) and invasive (China) countries. Indeed, our findings showed that the niche of *A. adenophora* was not equivalent in its native (Mexico) and invasive (China) countries, while the degree of niche overlap was low, indicating its niche has shifted in China, manifesting significant expansion of its climatic niche and potential geographical distribution. We elucidated the dynamics of the geographical distribution and dispersal of *A. adenophora* under the interaction of precipitation and temperature variables and offered a framework for preventing and controlling the spread of this IAP in China.

### 4.2 Niche dynamics of *A. adenophora*

We compared the niche dynamics of *A. adenophora*, including its climate niche and potential geographical distribution, between the native (Mexico) and invasive (China) countries. The niche overlap between Mexico and China appeared to be low, further elucidating that *A. adenophora* occupies a wider climate space in China than in Mexico. As such, the niche of *A. adenophora* is expanded in China compared with that in Mexico. Specifically, the climatic niche of *A. adenophora* in Mexico is a subset of that in China. Previous studies have compiled ample evidence of niche shifts of IAPs during the invasion process at the biogeographic scales. For instance, the native climate niche of *A. adenophora* in Mexico is not identical to any of its climate niches on other continents (Datta et al., 2019). In addition, the climate niches of invasive shrubs (*Ulex europaeus* L.) and *Ambrosia artemisiifolia* L. have expanded worldwide (Christina et al., 2019; Gallien et al., 2016). Likewise, the climatic niches of *Ligustrum lucidum* W.T.Aiton differed between its invasive and native zones (Dreyer et al., 2019). Furthermore, the climatic niche of 65% out of 815 introduced terrestrial plants was markedly shifted (Zachariah Atwater & Barney, 2021). Taken together, these findings indicate that IAPs may undergo niche shifts upon successfully invading new zones. The niche expansion of *A. adenophora* observed in the present study was consistent with this hypothesis. On the contrary, according to some studies, niche shifts are rare among terrestrial IAPs, whilst most IAPs occupy conserved climatic niches. Niche conservatism of IAPs is considered common at different geographical spatial and climatic scales. For instance, according to a large‐scale test across Eurasia, North America, and Australia, a great majority of 50 terrestrial IAPs occupied conserved climatic niches (Petitpierre et al., 2012). Similarly, niche conservatism is documented in African woody flora (Gorel et al., 2022). Our findings showed that the climate niche of *A. adenophora* has been conserved during its early invasion process in China. In brief, based on the native climate niche, *A. adenophora* occupies a broader climate space in China, indicating that IAPs may not lose their native climate niche during invasion. Collectively, our findings support both niche shift hypotheses. The niche shift of IAPs is expected to affect SDM performance (Liu et al., 2020b). SDM predictions based on native climatic data may underestimate the niches of IAPs in their invasive zones (Qiao et al., 2019). Therefore, we used both equilibrium occurrence data and bioclimatic data of *A. adenophora* to model its potential geographical distribution in China and revealed a more comprehensive niche and invasion risk of this IAP in its invasive region.

### 4.3 Expansion of the geographical distribution of *A. adenophora* in China

*Ageratina adenophora* has rapidly spread in Southwest China. Our results indicated that the earliest invasive zones of *A. adenophora* in China are located at the edge of the tropical zone. With continued spread, *A. adenophora* occupied the entire climatic niche of the native zones (Mexico). According to a previous study, *A. adenophora* spread to the subtropical zone after 1990 (Wang & Wang, 2006). During the early 21st century, the geographical distribution of *A. adenophora* in China was seemingly unsaturated (Wang et al., 2011). Here, we confirmed this hypothesis based on the analysis of our survey data of *A. adenophora* in China, which revealed that this IAP has spread to higher latitudes and warmer zones as opposed to its distribution before the 21st century. Moreover, the areas of the potential geographical distribution of Crofton weed harbor many rivers and water systems. *Ageratina adenophora* seeds can drift naturally over long distances along rivers. Therefore, river and water systems act as significant drivers of the dispersal of this IAP. The current geographical distribution of *A. adenophora* in China has reached the northern boundary of its predicted geographical distribution. Further, the geographical distribution of *A. adenophora* may expand to Southeast China, which has suitable climatic conditions for colonization. To date, regional studies in Nepal (Poudel et al., 2020), India (Chaudhary et al., 2021), and South Africa (Tererai & Wood, 2014) have predicted that the geographic distribution of *A. adenophora* will expand under climate change. Therefore, climate warming appears to be conducive to the survival of *A. adenophora*, augmenting its global invasion risk. Since the 1940s, *A. adenophora* has spread northwards to higher‐latitude zones in China. With future climate change, the geographical distribution of IAPs will rapidly spread to higher latitude zones (Petitpierre et al., 2016). The epigenetic modifications might lead *A. adenophora* to spread into the cooler zones of China. For instance, *A. adenophora* preferred lower temperatures in the invasive zones compared with the native zones (Tererai & Wood, 2014). The population of *A. adenophora* in China exhibited genetic variations (Huang et al., 2009), and the phenotypic plasticity has enabled *A. adenophora* to invade a wide range of habitats in China (Datta et al., 2017). The genetic variations of *A. adenophora* might have a great impact on the niche expansion of it in China. Our results support this conclusion regarding the global‐warming‐driven and genetic variations shift in the distribution of IAPs. Temperature and precipitation variables are pivotal factors determining the success of the invasion of IAPs (Fang et al., 2021; Qiu et al., 2018). The spatiotemporal distribution pattern of *A. adenophora* is shaped by the interaction between biological characteristics and multiple environmental variables, such as temperature and precipitation. Previous studies have identified the minimum temperature of the coldest month (Bio6) as a significant variable determining the geographical distribution of *A. adenophora* in Nepal (Poudel et al., 2020). Consistently, our results showed that the geographic distribution of *A. adenophora* in China is shaped by the combination of precipitation and temperature variables.

Overall, the present study proposes a novel approach to assess niche expansion, potential geographical distribution, and spread of *A. adenophora* based on occurrence records and bioclimatic data in the native (Mexico) and invasive (China) countries. Our findings offer insights into the heterogeneity of *A. adenophora* niches between its native and invasive countries. To the best of our knowledge, the present study is the first to analyze niche shifts of *A. adenophora* in China. Our work can serve as a reference for further prevention and control of *A. adenophora* spread, as well as the analysis of niche dynamics of other IAPs in China.

In conclusion, the niche dynamics of *A. adenophora* can be attributed to changes in its climate niche and geographical distribution between the native and invasive zones. Our optimal MaxEnt model showed good prediction accuracy. Furthermore, a greater extent of the climate niche space of *A. adenophora* in China is unoccupied than that in Mexico. The niche overlap of *A. adenophora* between its invasive (Mexico) and native (China) countries is low, indicating that the climate niche of *A. adenophora* has undergone significant alteration during its invasion process in China. Specifically, the invasive populations of *A. adenophora* exhibit better suitability to lower temperatures and higher precipitation levels than its native populations. The areas of the potential geographical distribution of *A. adenophora* are concentrated in the tropical and subtropical zones of China. Our findings support the hypothesis of niche shifts. As such, the native climate niche of *A. adenophora* has been preserved and has further expanded during its invasive processes in China. The observed niche dynamics of *A. adenophora* indicate its exacerbated invasion risk in China. Therefore, more attention should be paid to regular monitoring in potential geographical distribution areas where *A. adenophora* has not been colonized as yet. Such efforts can provide an early warning to prevent the further spread of this IAP and its consequent adverse effects in China. In the future, we will focus on the modeling integration for species distribution models, experimental research, and population genetics for *A. adenophora* so as to better explain the mechanism for its niche expansion.

## AUTHOR CONTRIBUTIONS

**Xiaoqing Xian:** Conceptualization (equal); data curation (equal); formal analysis (equal); investigation (equal); methodology (equal); resources (equal); software (equal); supervision (equal); validation (equal); visualization (equal); writing – original draft (equal); writing – review and editing (equal). **Haoxiang Zhao:** Conceptualization (equal); data curation (equal); formal analysis (equal); investigation (equal); methodology (equal); resources (equal); software (equal); supervision (equal); validation (equal); visualization (equal); writing – original draft (equal); writing – review and editing (equal). **Rui Wang:** Methodology (equal); writing – review and editing (equal). **Hongbin Zhang:** Formal analysis (equal); supervision (equal); validation (equal). **Baoxiong Chen:** Investigation (equal); methodology (equal); validation (equal). **Wanxue Liu:** Data curation (lead); funding acquisition (lead); project administration (lead); resources (equal); supervision (lead); writing – review and editing (equal). **Fanghao Wan:** Funding acquisition (equal); project administration (equal); writing – review and editing (equal).

## CONFLICT OF INTEREST

No conflicts of interest are declared.

## Supporting information


Appendix S1:

## Data Availability

The occurrence data are available at https://datadryad.org/stash/share/xGzpRnFIB0DsO8M9vrwHl018S5r5KO2Nu7cIVH2BM3I. The 19 bioclimatic variables are downloaded from the WorldClim database (http://www.worldclim.org/).
