# Supplementary material for: Evidence of the niche expansion of crofton weed following invasion in China
Source: Ecol Evol. 2023 Jan 6;13(1):e9708. doi: 10.1002/ece3.9708 (PMC9817199; doi:10.1002/ece3.9708)
Supplement: Supplementary file 1 — Appendix S1: [file ECE3-13-e9708-s001.docx]

**Appendices**


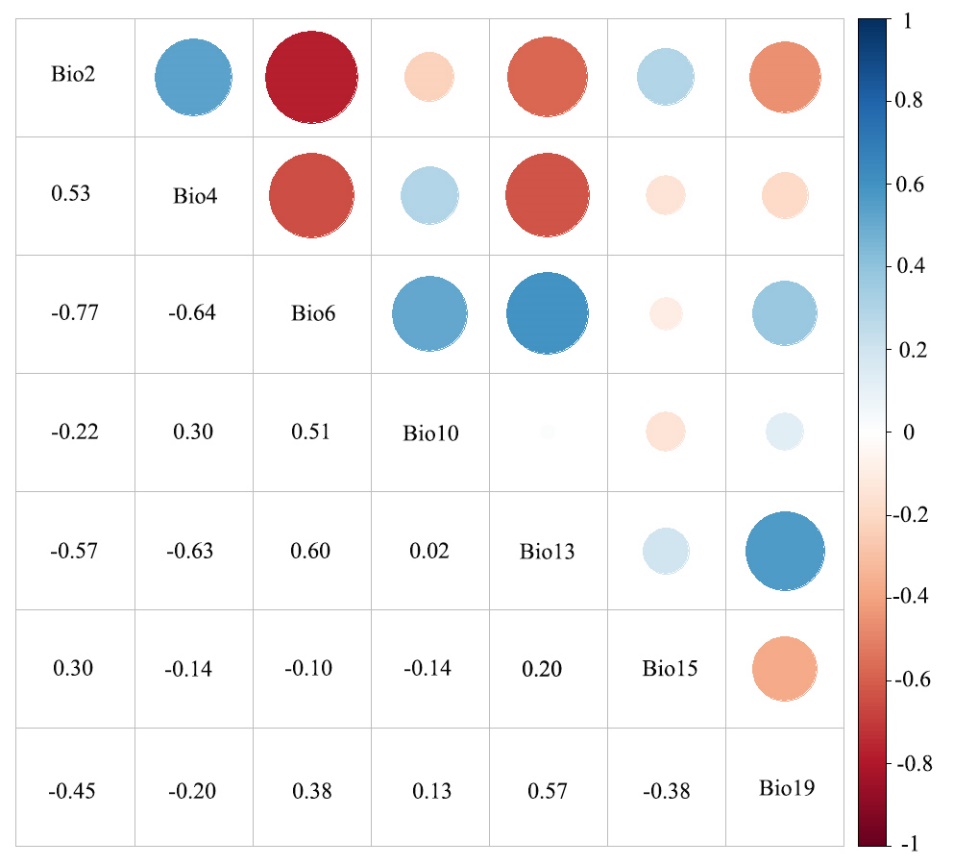


**Figure S1** Pearson correlation coefficients for the seven environmental variables retained for MaxEnt modeling in Mexico.


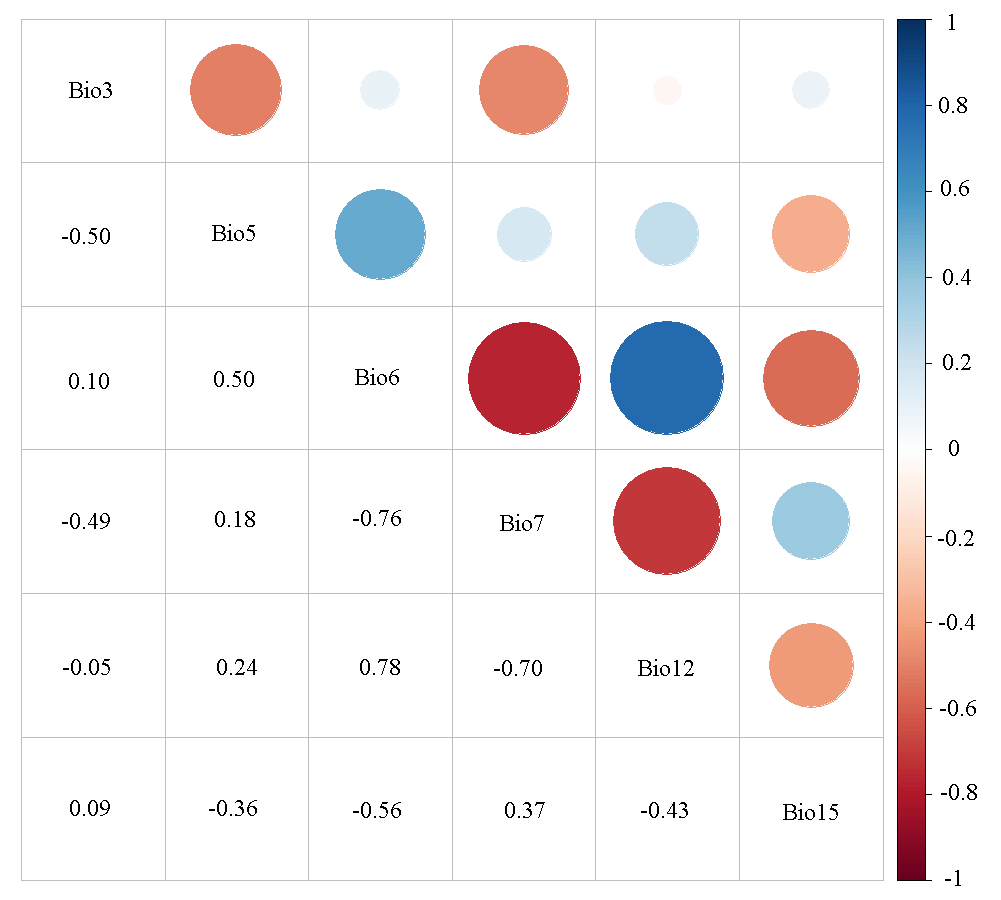


**Figure S2** Pearson correlation coefficients for the six environmental variables retained for MaxEnt modeling in China.

**
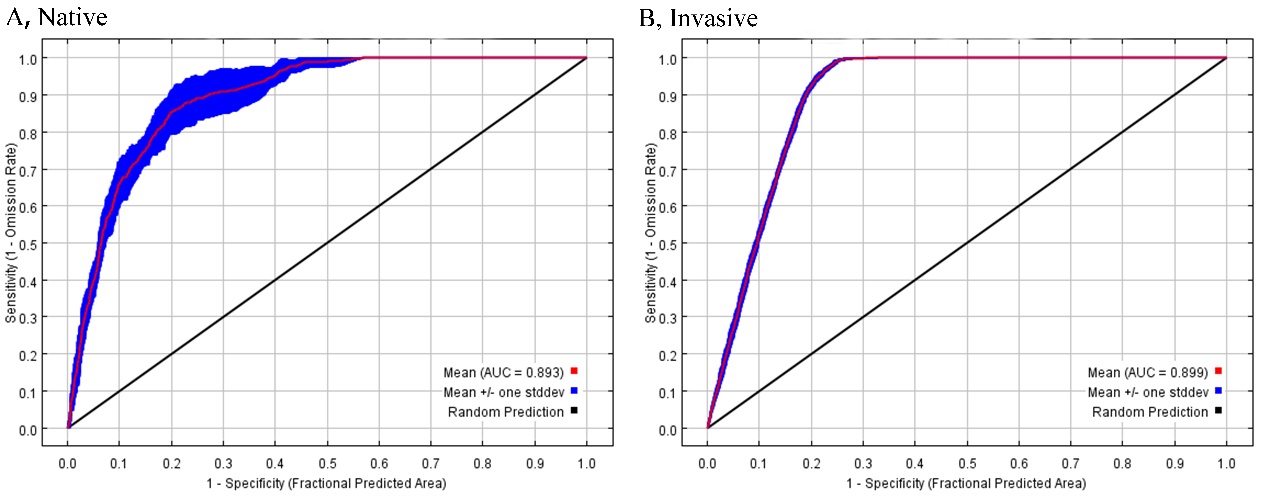
**

**Figure S3** The area under the receiver operating characteristic curve (AUC) values of the optimal model. A: Native (Mexico), B: Invasive (China).

**Table S1** Environmental variables related to the distribution of *Ageratina adenophora*

| Variables | Description | In model (Yes/No) | Unit |
| --- | --- | --- | --- |
| Bio1 | Annual mean temperature | No | ℃ |
| **Bio2** | **Mean diurnal range** | **Yes** | **℃** |
| Bio3 | Isothermality | No | - |
| **Bio4** | **Temperature seasonality** | **Yes** | **℃** |
| Bio5 | Maximum temperature of the warmest month | No | ℃ |
| **Bio6** | **Minimum temperature of the coldest month** | **Yes** | **℃** |
| Bio7 | Temperature annual range | No | ℃ |
| Bio8 | Mean temperature of the wettest quarter | No | ℃ |
| Bio9 | Mean temperature of the driest quarter | No | ℃ |
| Bio10 | Mean temperature of the warmest quarter | No | ℃ |
| Bio11 | Mean temperature of the coldest quarter | No | ℃ |
| **Bio12** | **Annual precipitation** | **Yes** | **mm** |
| Bio13 | Precipitation of the wettest month | No | mm |
| **Bio14** | **Precipitation of the driest month** | **Yes** | **mm** |
| Bio15 | Precipitation the seasonality | No | - |
| Bio16 | Precipitation of the wettest quarter | No | mm |
| Bio17 | Precipitation of the driest quarter | No | mm |
| Bio18 | Precipitation of the warmest quarter | No | mm |
| **Bio19** | **Precipitation of the coldest quarter** | **Yes** | **mm** |
